# DualDiscWaveGAN-Based Data Augmentation Scheme for Animal Sound Classification

**DOI:** 10.3390/s23042024

**Published:** 2023-02-10

**Authors:** Eunbeen Kim, Jaeuk Moon, Jonghwa Shim, Eenjun Hwang

**Affiliations:** School of Electrical Engineering, Korea University, 145 Anam-ro, Seongbuk-gu, Seoul 02841, Republic of Korea

**Keywords:** animal sound classification, deep learning, data augmentation, GAN

## Abstract

Animal sound classification (ASC) refers to the automatic identification of animal categories by sound, and is useful for monitoring rare or elusive wildlife. Thus far, deep-learning-based models have shown good performance in ASC when training data is sufficient, but suffer from severe performance degradation if not. Recently, generative adversarial networks (GANs) have shown the potential to solve this problem by generating virtual data. However, in a multi-class environment, existing GAN-based methods need to construct separate generative models for each class. Additionally, they only consider the waveform or spectrogram of sound, resulting in poor quality of the generated sound. To overcome these shortcomings, we propose a two-step sound augmentation scheme using a class-conditional GAN. First, common features are learned from all classes of animal sounds, and multiple classes of animal sounds are generated based on the features that consider both waveforms and spectrograms using class-conditional GAN. Second, we select data from the generated data based on the confidence of the pretrained ASC model to improve classification performance. Through experiments, we show that the proposed method improves the accuracy of the basic ASC model by up to 18.3%, which corresponds to a performance improvement of 13.4% compared to the second-best augmentation method.

## 1. Introduction

Animal sound classification (ASC) plays an important role in wildlife monitoring systems, as it automatically identifies animal categories by sound [1]. ASC is a particularly useful tool for cases where visual identification is difficult, such as small animals, nocturnal animals, and camouflaged animals. Recently, deep learning-based models such as convolutional neural networks (CNNs) have been widely used in ASC [2,3] as well as other signal processing applications. Although they show excellent classification performance by using temporal and frequency characteristics suitable for sound classification, their performance is greatly affected by the quality and quantity of the animal sound data used for learning. Collecting a sufficient amount of high-quality animal sound data is costly and time-consuming. If the amount of animal sound data for training a deep learning model is insufficient, then the classification performance can be significantly degraded due to improper learning [4]. This data shortage problem is particularly acute for animals that are difficult to observe, such as rare species.

To overcome this difficulty, data augmentation, which is a method of increasing the amount of data by transforming existing data in various ways, can be used [5]. Well-augmented data can be effectively used for model training, avoiding overfitting problems that result in poor classification performance. However, most species have different sound features in pitch and speed, and traditional data augmentation methods such as pitch scaling and time stretching do not properly account for the unique characteristics of each species. This implies that existing augmentation methods require prior knowledge of the target animal sound and delicately select transformation operations. Otherwise, they may distort the feature information of animal sounds, resulting in poor classification performance.

A more recent trend to deal with the data shortage problem is to use generative adversarial networks (GANs) [6]. GAN is a data generative model consisting of two networks: a generator and a discriminator. Based on the adversarial learning process of these two networks, the GAN generates realistic virtual data by learning the distribution of the real data and is thus widely used for data augmentation in various domains such as image, time-series, and signal processing [7,8].

Despite the potential of GANs, there are some limitations to the direct application of traditional GAN-based data augmentation approaches to ASC:

1. In a multi-class environment, the GAN should be constructed for each animal sound class to be generated. This requires a huge amount of time and storage space as the number of target classes increase. Further, for classes with insufficient training data, such as rare animal sounds, the quality of the generated data can be very poor.
2. Animal sounds have complex patterns made up of multiple acoustic components such as frequency, duration and tempo. In order to generate realistic animal sound data, a GAN should consider not only the waveforms that represent the signal intensity over time, but also the spectrograms that contain the frequency-time features of the sound to capture periodic patterns. However, existing GAN-based augmentation methods only consider either waveforms or spectrograms to generate sound data [9,10].
3. Although GANs show good generative performance, their results are closely influenced by the quantity and quality of the real animal sound data used for training. In particular, as animal sounds are usually collected in an outdoor environment, the collected sounds may contain significant background noise, such as wind and rain, despite noise reduction operations. Because of these noises, GANs cannot effectively learn the characteristics of real animal sounds, resulting in virtual sounds that lack semantic information representing distinct animal sounds.

To address the aforementioned limitations, in this paper, we propose a novel class-conditional GAN-based animal sound data augmentation scheme for ASC. Our scheme consists of two stages: GAN-based data generation stage and confidence-based data selection stage. In the first stage, we generate realistic sounds of multiple animal classes using a class-conditional GAN structure. Besides the usual single generator, the class-conditional GAN has two discriminators to process waveforms and spectrograms, respectively. In the second stage, based on the confidence score calculated by the pretrained ASC model for the generated sound data, the top-ranked data are selected and used for augmenting the training data. Such data can be effectively used for training because they well represent class-specific semantic information. To demonstrate the effectiveness of our scheme, we compare the quality and diversity of the generated sound data with those generated by other conditional generation models. We also compare the ASC performance of the proposed scheme and that of other popular augmentation methods in terms of traditional classification metrics and demonstrate the robustness of our system.

The contributions of this paper are summarized as follows:

- We propose a novel two-stage sound data augmentation scheme using a class-conditional GAN to solve the data shortage problem in ASC.
- We present an effective way to consider both the waveform and the spectrogram of sound to plausibly generate animal sound data. In addition, we propose a data selection method for augmentation from the generated data to improve ASC performance.
- We compare the ASC performance of the proposed scheme with other popular data augmentation methods through various experiments on real-world audio datasets of bird and frog species. Furthermore, we validate the class-specific and aggregate generative capability of the proposed scheme.

The remainder of this paper is organized as follows: We first introduce the backgrounds of GAN, ASC, and data augmentation in Section 2. In Section 3, we briefly describe the proposed data augmentation scheme for ASC. The experimental settings and results are presented in Section 4 and Section 5, respectively. Finally, the major conclusions drawn from the study results are elucidated in Section 6.

## 2. Related Work

This section first provides a basic overview of GAN, and then briefly introduces various previous studies on sound classification.

### 2.1. Overview of GAN

GAN is a data generation model based on the ideas of game theory [6], which can generate realistic virtual data as output by learning the distribution of real data. GAN consists of generator and discriminator. The goal of the generator is to generate virtual data that resemble real data to deceive the discriminator into determining the generated data as real data. The discriminator incorporates both the virtual data of the generator and the real data as inputs and determines whether the input data are real or not. Because of these conflicting goals, both networks are competitively trained simultaneously; this process can be expressed by Equation (1).

(1) $$V\left(D,\;G\right)=E_{x\sim p_{r}}\left[\log D\left(x\right)\right]+E_{z\sim p_{z}}\left[\log\left(1-D\left(G\left(z\right)\right)\right)\right]$$

Here, *x* and *z* refer to the data taken from the real data distribution *p_r_* and the latent variable obtained from the latent distribution *p_z_*, respectively; *G*(*z*) is the virtual data obtained from the generator, which utilizes *z* as an input; and *D* is the function of the discriminator that outputs 1 or 0, when the given data are real or fake, respectively. The generator aims to maximize the function *V*(*D*, *G*) such that *D*(*G*(*z*)) becomes 1, while the discriminator aims to minimize *V*(*D*, *G*) so that *D*(*G*(*z*)) becomes 0. Because of such conflicting objectives, it is difficult for the generator and discriminator to achieve their own goals. However, at the end of the training of the GAN, the generator can generate realistic virtual data that cannot be distinguished by the discriminator.

### 2.2. Data Augmentation for Sound Classification

In recent years, deep learning technology has shown remarkable progress in various fields. For instance, CNN-based models performed well in the ASC task due to their capabilities such as feature extraction for sound classification, complex pattern recognition, and robustness to noise. Specifically, Nanni et al. [11] proposed an ensemble model of CNNs such as AlexNet, GoogleNet, and ResNet for automated animal audio classification. They first converted the animal sound waveform into various visual features such as spectrograms and harmonic images, and then constructed CNN-based ensemble models using these different visual features. Through extensive experiments on several animal audio datasets, they demonstrated that an ensemble model composed of CNNs can perform robust and generalizable audio classification. In order to utilize more diverse features, multi-stream-based techniques have been proposed, where different types of data such as visual features and acoustic features are used together as inputs. For instance, Wu et al. [12] proposed a dual attention matching method to classify and localize the category of video segments composed of visual and audio data. This method combines related features of images and waveforms using an attention mechanism. They showed that their method outperforms other multi-stream methods in classification and localization problems, such as the audio-visual event localization task. In a similar context, Xie et al. [13] presented a CNN-based ASC scheme that utilizes both waveforms and mel-spectrograms together. Based on the sensitivity of one-dimensional (1D)-CNN depending on a waveform to background noise and class imbalance, they investigated various combinations of three CNN architectures and four loss functions. Then, they showed that a combination of 1D-2D-CNN and focal loss, which fuses waveform and spectrogram features, is most effective for classifying both Australian and Brazilian frog calls.

To achieve good classification performance, CNN-based deep learning models require a large amount of high-quality animal sound data for training. However, constructing a large-scale sound dataset for rare animals is quite challenging as collecting sound data from these animals in real life is time-consuming and costly. This limitation can be mitigated via various sound data augmentation methods [14], which can be largely divided into two types: waveform augmentation and spectrogram augmentation. For waveform augmentation, methods such as pitch shifting, time stretching, and noise addition are commonly used to increase the frequency or temporal diversity of sound data [5]. For example, pitch shifting raises or lowers the pitch of a sound waveform by a preset range. Similarly, time stretching increases or decreases the speed of a waveform by a preset value. Noise addition mixes the target waveform with various types of noise, such as white noise or background noise [15]. Mushtaq and Su [16] used these waveform augmentation methods to train a CNN model for classifying environmental sounds such as dog barking and drilling. They showed that augmented sound data can significantly improve classification accuracy by preventing the CNN model from overfitting small amounts of training data. Meanwhile, spectrogram augmentation was suggested more recently for sound data augmentation. For instance, Park et al. [17] introduced frequency and time masking methods, motivated by the idea that deep networks should be robust against a partial loss of frequency or time information. These two methods remove the spectrum information by randomly masking the frequency rows or time columns, respectively, from the spectrogram. They demonstrated that their augmentation methods could significantly improve the accuracy of human speech recognition. Nanni et al. [5] performed extensive experiments on bird and cat sound datasets and found that most waveform and spectrogram data augmentation methods are beneficial for training CNN models, although some augmentation methods are useless or even degrade ASC performance. This indicates that the characteristics of the domain data should be considered when selecting an augmentation method and determining its transformation parameters.

Due to the limited availability of traditional augmentation methods, GAN-based models have attracted considerable attention as data augmentation tools in the signal data domain. For instance, Esmaeilpour et al. [18] suggested a weighted cycle-consistent GAN (WCCGAN) for spectrogram augmentation. Their method transfers the structural features of source spectrogram to target spectrogram, generating deformed data. They showed that the accuracy of two classifiers trained with augmented data by WCCGAN improved significantly on four environmental sound datasets. Madhu and Suresh [19] developed an unconditional GAN-based augmentation model by adding two layers and one stable loss function to WaveGAN [20] to generate longer virtual waveforms suitable for representing environmental sounds. However, this approach requires a class-specific generative model to generate class-specific data properly in a multi-class environment. Consequently, as the number of sound classes increases, the time and effort required for model construction also increase.

This problem can be addressed by using conditional GANs that generate multiple class data within one unified model. For instance, Jayalakshmy et al. [21] used a conditional GAN for respiratory waveform augmentation. They combined a 1D GAN with a standard conditional GAN (cGAN) [22], whose generator and discriminator receive conditions via embedding layers and concatenation operations. Similarly, Seibold et al. [23] proposed a data augmentation scheme based on the conditional Wasserstein GAN with gradient penalty (WGAN-GP) [24] for clinical audio classification. This scheme generated log-mel spectrograms as input to a ResNet-based classifier and achieved better classification performance compared to other classic signal augmentation methods. Shao et al. [25] suggested an auxiliary classifier GAN (ACGAN) [26] for data augmentation to diagnose machine faults. To generate class-wise sensor data, they used the work type as a class condition for training the ACGAN. Through experiments, they showed that the ACGAN-based augmentation strategy can effectively compensate for imbalanced datasets and generate convincing sensor signal data.

However, these cGAN-based approaches use a single discriminator that verifies the realness of virtual data in only one of the waveform and spectrogram [9,10]. This restricts the learning of discriminative features of different classes of sounds, making it difficult to reproduce some animal sounds with subtle differences. Furthermore, they cannot handle the case where virtual data with ambiguous characteristics is generated due to the influence of environmental noise included in real data. To overcome these limitations, we propose two discriminators to simultaneously process the waveforms and spectrograms of real animal sounds and a data selection technique to filter out ambiguous virtual animal sounds.

## 3. Method

**Figure 1:**
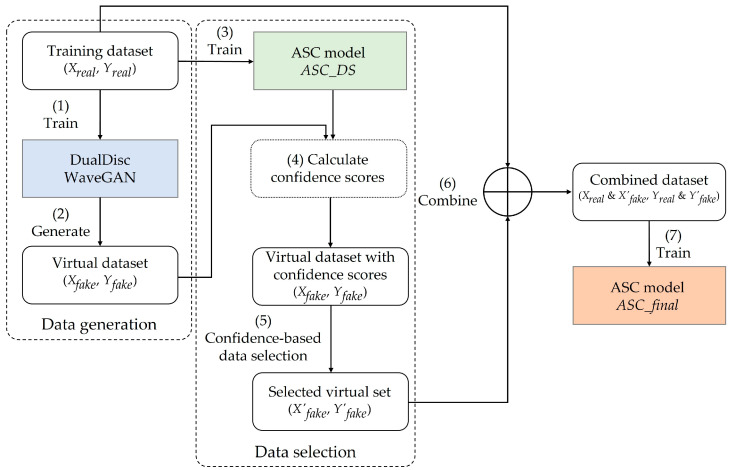
Augmentation process of the proposed scheme.

This section describes the proposed scheme for data augmentation in more detail. As shown in Figure 1, the scheme consists of two stages: animal sound data generation and data selection. In the sound generation stage, DualDiscWaveGAN is trained using a training dataset (*X_real_*, *Y_real_*) to generate a virtual dataset (*X_fake_*, *Y_fake_*). In the data selection stage, an ASC model, named *ASC_DS*, is trained using the original training data to calculate confidence scores for the generated data. Then, data with a high confidence score are selected among the generated data. Finally, the selected dataset (*X*′*_fake_*, *Y*′*_fake_*) combined with the original training data are used for training to construct the final ASC model, named *ASC_final*.

### 3.1. DualDiscWaveGAN

As mentioned earlier, although existing GAN models have shown the potential of sound data augmentation [19], their effectiveness is quite limited in multi-class applications such as ASC. To overcome this, we propose DualDiscWaveGAN, a new GAN model for conditional animal sound generation. To mitigate the problem of poor generative quality due to lack of training data for classes, we train a single GAN using sounds from multiple animals belonging to the same order in biological taxonomy (e.g., anura or passeriformes). Because the sounds produced by these animals are more similar than those of the other animals in other orders, their sounds will help the model to learn the common features. In addition, by embedding a class label representing the animal of that sound into a GAN, the GAN can effectively generate virtual animal sounds containing unique characteristics of a given class. For generating waveforms, we used WaveGAN [20], which can generate a variety of perceivable sounds up to a duration of 1 s (16 kHz), such as drum and piano sounds. However, the waveform discriminator in the existing WaveGAN only determines if the generated waveform is realistic. This may not be sufficient to reproduce the distinct characteristics of each animal sound, because the corresponding waveform is not suitable for representing the frequency characteristics of animal sounds. Therefore, we added a spectrogram discriminator based on SpecGAN [20], which examines whether the virtual spectrogram converted from the virtual waveform by short-time Fourier transformation (STFT) is realistic. By using these two discriminators, it is possible to reflect their characteristics in the process of animal sound generation.

**Figure 2:**
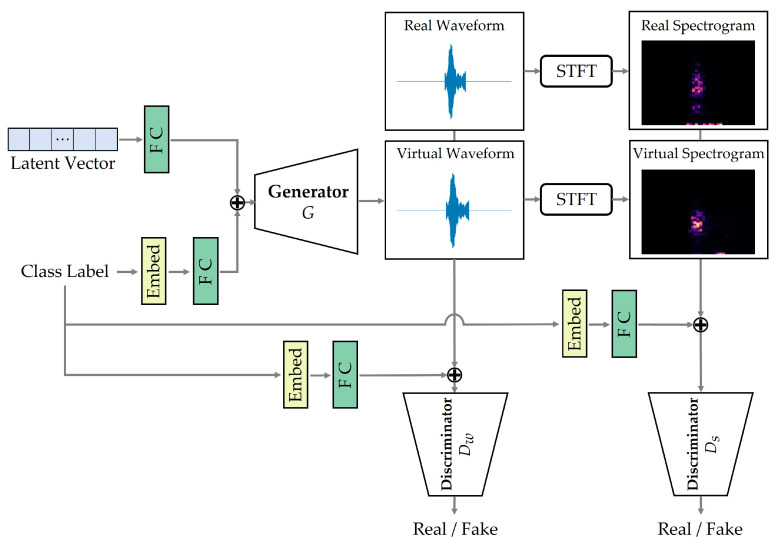
Overall architecture of DualDiscWaveGAN. FC denotes the fully connected layer; Embed denotes the embedding layer; STFT denotes short-time Fourier transform; *D_w_* denotes the waveform discriminator; and *D_s_* represents the spectrogram discriminator.

Figure 2 shows the overall architecture of our DualDiscWaveGAN. The model consists of one generator that produces a specific waveform of a given label and two discriminators that determine the authenticities of the input waveform and spectrogram with the given label. The generator accepts a random latent vector *z* from a normal distribution between 0 and 1 and a class label *c*, which indicates the particular class it wants to generate, as the input. To analyze the class label, we used an embedding layer that transforms the input label into an embedding vector, which is then trained to represent the unique characteristics of animal sounds that correspond to a specific label when learning multiple animal sound data. Then, the input latent and embedding vectors are combined to form feature vectors through fully connected layers and channel-wise concatenation. The generator converts these feature vectors from low-resolution to high-resolution vectors using multiple transposed convolutions and produces a raw waveform of 16,384 samples [20] corresponding to the given label. Similar to the generator, in the discriminators, the input label is converted into an embedding vector and then combined with the input waveform or spectrogram through the fully connected layers and channel-wise concatenation. Then, the discriminators extract the features of the input waveform or spectrogram through multiple convolution layers to determine the authenticity of the input data.

### 3.2. Adversarial Loss

In this section, we describe the adversarial loss used to train the proposed DualDiscWaveGAN. For a stable training, we use the loss function of the WGAN-GP [24] as the adversarial loss; it consists of the loss function of WGAN (first two terms) and the gradient-penalty loss (last term). Unlike vanilla GAN’s loss function, which uses a log function that is the same as that shown in Equation (1), the WGAN uses a simple difference between the discriminator outputs of the real and generated data. The loss function of WGAN provides a stable convergence of the loss by mitigating the mode collapse, which cannot precisely reproduce the learned data distribution and generate various sample examples. Meanwhile, the GP loss enables the discriminator to become a 1-Lipschitz function by adjusting the gradient norm of the discriminator for random data $\hat{x}\sim p_{\hat{x}}$, where $p_{\hat{x}}$ is the generated virtual data distribution. As a result, the GP loss contributes to the stable adversarial training by preventing gradient explosions in the generative models. Equation (2) represents the loss function of WGAN-GP:

(2) $$L_{adv}\left(D,\;G\right)=E_{z\sim p_{z}}\left[D\left(G\left(z\right)\right)\right]-E_{x\sim p_{r}}\left[D\left(x\right)\right]+\lambda E_{\hat{x}\sim p_{\hat{x}}}\left[{({\left\|\nabla_{\hat{x}}D\left(\hat{x}\right)\right\|}_{2}-1)}^{2}\right]$$

Here, $p_{r}$ and $p_{z}$ are the distributions of the real data and latent vector, respectively; and λ represents a coefficient of the GP.

As described earlier, our generation model has two discriminators to validate the input waveform and its spectrogram. Therefore, we compute the adversarial losses for each discriminator as well as calculate each loss function using the real and virtual data with the same class for conditional generation. Equation (3) defines the final loss function used in DualDiscWaveGAN:

(3) $$L\left(D_{w},D_{s},\;G\right)=L_{adv}\left(D_{w},\;G\right)+L_{adv}\left(D_{s},\;G\right)$$

Here, *D_w_* and *D_s_* are the waveform and spectrogram discriminators, respectively.

### 3.3. Confidence-Based Data Selection

Animal sounds generated by DualDiscWaveGAN can improve the generality of the training data and avoid overfitting that causes ASC performance degradation. However, since the animal sounds recorded in the real world and used for GAN training often include various background noises, the GAN may generate virtual sounds that lack the semantic information of real animal sounds. In other words, some virtual sounds that closely mimic the features of real animal sounds help improve the ASC performance, while virtual sounds that deviate significantly from real sounds can act as outliers and degrade the ASC performance [4]. Therefore, we calculate the classification confidence of the virtual data and select data with high confidence scores for the data augmentation step.

We first train an ASC model *ASC_DS* using the real data (animal sound data) *X_real_* and their categories (animal class label) *Y_real_*, based on the cross-entropy loss. This ASC model can distinguish the unique features of each real animal sound, and hence, a clearer semantic information of the input sound will aid the model in correctly classifying the corresponding class. Therefore, we use *ASC_DS* to infer the probability of the generated virtual data *X_fake_* for each class, and based on this probability, we determine the confidence score for the virtual label *Y_fake_*. If the virtual data have a high confidence score, then they have characteristics similar to those of the real data. For each class, we select the virtual data with high confidence scores and use them to augment the training data for the final ASC model *ASC_final,* as shown in Figure 1. These training data are then used to train the final ASC model.

## 4. Experiments Setup

To verify the effectiveness of the proposed scheme, we conducted extensive experiments; first, we evaluated the generative capacity of the proposed scheme, and then examined the effectiveness of our data augmentation method by assessing the ASC performance.

### 4.1. Datasets

In this study, we considered two real-world animal sound datasets as follows.

- North American Bird Species (NA birds): This audio dataset, introduced by Zhao et al. [27], contains audio recordings of eleven bird species commonly observed in North America. The species are *Cyanocitta cristata*, *Melospiza melodia*, *Cistothorus palustris*, *Geothlypis trichas*, *Spizella passerine*, *Setophaga aestiva*, *Ardea herodias*, *Corvus brachyrhynchos*, *Bombycilla cedrorum*, *Haemorhous mexicanus*, and *Passerina cyanea*. All the recordings were collected from the Xeno-canto archive (https://xeno-canto.org/, accessed on 10 September 2022) and split into segments representing short songs or calls. The sounds are as varied as the mixed tones and partly contain background noise. In our experiments, we used 2515 segments from 10 birds, excluding *Ardea herodias*, which are classified in a different order in the biological classification.
- South Korean Frog Species (SK frogs): This dataset contains 16,245 audio segments collected from five species of anuran living in South Korea. The species are *Kaloula borealis*, *Bombina orientalis*, *Hyla suweonensis*, *Pelophylax nigromaculatus*, and *Hyla japonica*. All the anuran sounds were recorded in their habitats under the following conditions: sample rate of 44.1 kHz, single channel, and 16-bit resolution. Because many recorded sounds substantially overlap with other animal sounds and background noises, we divided all the recordings into multiple segments by using the end-point detection method [28], and removed the segments that were heavily intermingled with sounds from other species or loud noises.

For both the datasets, we equally resampled all the sound segments at a sample rate of 16 kHz and padded them to ensure a one-second length.

In this study, we used two experimental protocols to construct the training and test datasets for ASC: random separation and regional separation. The random separation protocol is one of the most common evaluation methods in the ASC field. It performs a stratified random sampling by dividing the entire dataset into training and test datasets; this ensures that each class has the same percentage of audio segments for both training and test datasets [27]. We randomly split each dataset into a training dataset (70%), a validation dataset (10%) and a test dataset (20%), then repeated the random separation and ASC evaluation five times and calculated their average.

By contrast, collecting animal sounds from diverse regions in the real world is arduous. In this case, the regional separation protocol can be used to validate the data augmentation method and ASC model. Therefore, we split the SK frogs dataset into a training dataset and a test dataset based on the regions from where the data were collected. As the NA birds dataset does not provide region information for the collected data, we conducted regional separation only for the SK frogs dataset. In this protocol, we constructed the validation dataset by randomly selecting 10% data from the training dataset.

### 4.2. Evaluation Metrics

One effective and practical way to evaluate the quality of the generated sounds is subjective evaluation by listeners [29], although it is a time-intensive process that requires participants with expertise in the field (e.g., expertise to distinguish ambiguous sounds of animal species). Therefore, we consider the following objective evaluation metrics to assess the quality of the generated sounds.

#### 4.2.1. Quality Evaluation Metrics

Fréchet inception distance (FID) [30], which is a representative evaluation metric used to assess the fidelity of the data generated by GANs, indicates the distance between two feature sets sampled from the real and virtual data distributions. Therefore, it represents the statistical similarity between them and can be defined by Equation (4) below:

(4) $$FID\left(R,V\right)={\left\|\mu_{R}-\mu_{V}\right\|}^{2}+Tr\left(\sigma_{R}+\sigma_{V}-2{\left(\sigma_{R}\sigma_{V}\right)}^{1/2}\right)$$

Here, *R* and *V* denote the real and virtual feature vector sets, respectively; $\mu_{R}$ and $\mu_{V}$ represent the means of the real and virtual features, respectively; *Tr* is the trace of the matrix; and $\sigma_{R}$ and $\sigma_{V}$ are the covariance matrices of the real and virtual features, respectively. A lower FID score indicates a better quality of the virtual data. Here, the feature vectors can be extracted from the intermediate layers of a pretrained classifier, and accordingly, we use a pretrained ASC model and convert the input waveforms to log-spectrograms similar to the process reported by Engel et al. [31].

However, the original FID metric was proposed for unconditional GANs; thus, we use its extended version, Intra-FID [32] for our conditional GAN. The FID is calculated using the data from all classes, whereas Intra-FID is calculated using only class-specific data. Therefore, by using both the metrics, we can capture the overall and class-wise generative performance of the conditional GAN.

#### 4.2.2. Diversity Evaluation Metrics

Another important metric for generative models is the number of statistically-different bins (NDB), which indicates the diversity of the generated data [33]. This metric NDB yields a score for diversity as well as captures the mode collapse, and to calculate it, real samples are clustered into *K* different bins using *K*-means algorithms. Subsequently, virtual samples are assigned to the closest bin based on the L2 distance to each bin centroid, and finally, a two-sample test is performed on each bin. Bins with significantly different bin proportions (the ratio of samples assigned to the bin to the total samples) of the real and virtual samples are calculated as the NDB scores. Therefore, a lower NDB score is indicative of a better diversity.

Similar to the process followed by Liu et al. [34], we extend the NDB to NDB_all-classes_ and NDB_class-wise_ to validate the diversity of the conditional generation with a higher accuracy. Here, NDB_all-classes_ considers the data from all the classes, whereas NDB_class-wise_ considers data by class, implying that the number of class-wise real data is much smaller than those of the all-class data. Therefore, we set *K* = 20 and 100 for NDB_class-wise_ and NDB_all-classes_, respectively, and performed all the NDB evaluations using log-spectrograms.

### 4.3. Implementation Details

In this section, we describe the details of the experimental setup used in our study. First, we trained the generator and the two discriminators of DualDiscWaveGAN using the Adam optimizer with a learning rate, *β*_1_, and *β*_2_ of 0.0001, 0.5, and 0.9, respectively. In addition, we used a GP coefficient (λ) of 10 for a stable training. These hyperparameter settings were selected according to [20]. The discriminator updates were performed five times per generator update, as suggested by Gulrajani et al. [24]. We performed up to 10,000 iterations for training, and stopped the training when the FID score of the validation dataset showed the best performance. Especially, we used spectrograms that were obtained by performing STFT (256 FFT size, 128 window size, 128 hop size) on the real and virtual waveforms as the input data for the spectrogram discriminator. Consequently, a waveform of 16,384 samples was converted into a 129 × 129 spectrogram.

To validate the effect of our data augmentation method on the ASC performance, we used ResNet-18 [35], a popular CNN-based image classification model, as the baseline model for the ASC. The model can effectively classify animal sounds by extracting high-level sound features based on deep layers and residual connections. Since the spectrogram is a popular handcrafted feature in sound classification [11], we used it as an input to the ResNet model. We performed STFT on the waveforms to obtain spectrograms, scaled their amplitudes logarithmically, and normalized them using the mean and standard deviation of each frequency bin obtained from the training dataset, as suggested in [20]. The ResNet model was trained up to 100 epochs on the training dataset, and the training was stopped upon loss convergence of the validation dataset. To evaluate the ASC performance of our ResNet model, we used the representative metrics of classification tasks: accuracy, precision, recall, and F1-score [36]. All the experiments were conducted using an Intel Core i7-9700 CPU, 32GB RAM, and NVIDIA GeForce GTX 1080ti GPU under a Python environment; all the models were implemented with PyTorch [37].

### 4.4. Comparative Data Augmentation Methods

To compare the data augmentation methods for ASC, we considered four traditional waveform augmentation methods and the latest two spectrogram augmentation methods, as shown in Table 1. For waveform augmentation, we used time stretching, pitch shifting, background noise addition, and white noise addition. The last two noise-addition methods mix the noises with the input waveforms to yield a signal-to-noise ratio of 0 dB. We collected various environmental noises, such as wind and rain sounds, from Freesound web DB (https://freesound.org/, accessed on 11 July 2022) and used them for the background noise addition. For spectrogram augmentation, we used frequency and time masking [17]. Following the conventional augmentation pipeline [5], whenever input data are provided to the ResNet model in the training loop, the aforementioned augmentation methods are applied with a probability of 50%. Conversely, our conditional GAN-based augmentation scheme generates 3 × *N* samples per class during the data generation, and selects *N* samples in order of confidence score during the data selection. Considering the size of the training dataset, we set *N* = 1000 and 3000 for the NA birds and SK frogs datasets.

## 5. Results and Discussion

This section describes the experiments performed in this study and the corresponding results. First, we evaluated the conditional generative capacity of the proposed scheme in terms of quality and diversity. Second, we validated the ASC effectiveness of our proposed scheme based on the aforementioned two dataset-separation protocols. Lastly, we investigated the components of our scheme through an ablation study.

### 5.1. Quality and Diversity Evaluation

To validate the conditional generative performance of DualDiscWaveGAN, we used cGAN [22], ACGAN [26], and Projection-cGAN [32], which are representative conditional GAN models widely used for conditional data generation, as the comparison models. Note that we extended them based on the WaveGAN architecture to generate waveforms effectively.

For the evaluation, we used the NA birds dataset, because bird species make a variety of sounds. If a generative model had learned such diverse data effectively, the features and distribution of the generated data should be similar to the actual data [38]. For a fair evaluation, all the conditional generative models were trained as described in Section 4.3, and the FID and NDB scores were recorded when the model showed the best FID score. Here, we used the entire real dataset to evaluate FID and NDB, and all the trained models generated the same number of virtual data as real data of each class.

**Figure 3:**
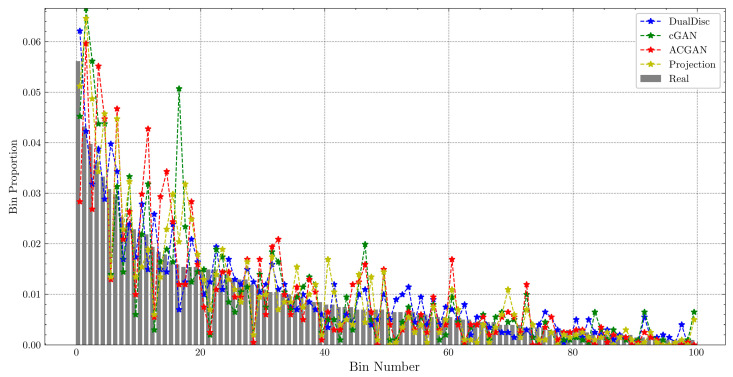
Bin proportions of the real and virtual data of all the classes. The number of bins, *K*, is 100. Projection indicates Projection-cGAN, and DualDisc indicates the proposed DualDiscWaveGAN. The bars represent the bin proportions of the real data, and the dash-dotted lines represent the bin proportions of the virtual data generated by each model.

Table 2 and Table 3 show the evaluation results for all the class and class-wise data, respectively. Table 2 shows that our DualDiscWaveGAN achieves the best performance in terms of FID and NDB. Especially, our scheme outperforms the other models in terms of NDB by a large margin, indicating that our scheme can generate a variety of virtual data that most closely resemble the real data. The NDB scores are depicted in Figure 3, wherein the bars indicate the bin proportions of the real data, and the dash-dotted lines indicate the bin proportions of the virtual data generated by each model. A comparison of the bin proportions of the real and virtual data reveals that cGAN and ACGAN over- or under-generate virtual data relative to the actual data in several bins. This indicates a lack of diversity in the learned distribution and can lead to problems such as overfitting during data augmentation. In contrast, our DualDiscWaveGAN generates the actual and virtual data almost equally.

To ascertain the conditional generative ability, we show the class-wise case of the comparative models in Table 3. Note that the last row of the table represents the averages of each class evaluation results. The comparison of the class scores of each model reveals that the conditional generative abilities of the models differ significantly. In the Intra-FID evaluation, DualDisc surpasses the other models in most classes, except for *Melospiza melodia* and *Cistothorus palustris*. Specifically, our model exhibits a better performance on several classes, whereas the other models show a relatively poor performance (e.g., *Geothlypis trichas* and *Spizella passerine*). In terms of NDB_class-wise_, ACGAN shows significant gaps between classes (e.g., *Cistothorus palustris* and *Haemorhous mexicanus*), whereas the overall gaps in the case of DualDisc are small. Because ACGAN relies on an auxiliary classifier for conditional generation, it seems to focus on learning a few types of easily distinguishable animal sounds using a classifier. On the other hand, DualDisc learns the waveform and spectrogram representations of animal sounds from each class, thereby achieving a higher performance. In summary, our model achieves the best averaged scores for both the metrics in the class-wise case. These results demonstrate that our DualDiscWaveGAN can reproduce realistic animal sounds with a high quality and diversity.

### 5.2. Comparsion with Different Data Augmentation Methods

In this section, we show the effectiveness of the proposed scheme based on ASC performance. We compared different data augmentation methods, based on the existing classification metrics, using a random separation protocol for both the NA birds and SK frogs datasets and a regional separation protocol for the SK frog dataset.

#### 5.2.1. ASC Experiment on the Random Separation Protocol

Table 4 shows the ASC performance of the ResNet model according to various augmentation methods applied on the NA birds dataset. In this experiment, the proposed scheme achieved the best performance in terms of all the metrics. Even without the data selection module, the scheme still improved the accuracy of the ResNet model by approximately 2.4%, thereby validating the effectiveness of DualDiscWaveGAN. Except for the time masking, all the other augmentation methods also slightly increased the classification accuracy of the ResNet model. In the case of background noise addition, the model training failed, despite using diverse augmentation parameters and background noise types. This observation suggests that the background noises cancel out the distinguishable features of each bird sound, thereby removing their semantic information. On the contrary, white noise addition improved the accuracy of the ResNet model by approximately 1.4%.

The experiments on the SK frogs dataset yielded a mean accuracy of 98.9% for the ResNet model over all the trials. Therefore, we did not perform further comparison experiments, because the performance was already satisfactory without data augmentation.

#### 5.2.2. ASC Experiment on the Regional Separation Protocol

In this experiment, we compared the ASC performance of the augmentation methods, applied on the SK frogs dataset, using the regional separation protocol. Table 5 reveals that for the frog sounds adopted from the test dataset consisting of unseen regions, the accuracy (65.8%) of the ResNet model is lower than that of the random separation protocol (98.9%) described in Section 5.2.1. This result is reasonable, considering that the collected frog sounds include different background noises depending on the data collection region, and thus, background and white noise additions were effective for training the ResNet model in most metrics, as they provided variations similar to regional environmental conditions. On the contrary, time stretching and pitch shifting degraded the performance of the ResNet model in most metrics. Since each frog has a distinctive sound with an absolute speed and pitch, these methods appear to distort the unique characteristics of the frog sounds. Similarly, frequency and time masking also analyzed the frequency and temporal features of the sounds without considering the characteristics of each frog sound, resulting in only a slight improvement or significant degradation of the ResNet performance. Despite the challenges of the existing augmentation methods for ASC, the proposed scheme enhanced the ResNet classifier performance in terms of all the metrics by large margins and achieved the best scores in terms of accuracy, precision, and F1-score.

### 5.3. Ablation Study

Through this experiment, we demonstrated the importance of each component in the proposed scheme. We performed the ASC experiment with the same setting as that mentioned in Section 5.2.2 using the SK frogs dataset. Note that the proposed scheme without a spectrogram discriminator and data selection is the same as a cGAN model as described in Section 5.1. As shown in Table 6, adding each component to our scheme steadily improved the performance of the ResNet baseline model in terms of all the metrics. In particular, the spectrogram discriminator exuded the greatest impact on the ASC performance. These results imply that when the two discriminators simultaneously consider the spectrogram and waveform features, realistic animal sounds are produced, and the ResNet model is trained more effectively and efficiently. In addition, performing data selection of the generated data in our scheme further improves the performance of the ResNet model.

## 6. Conclusions

In this paper, we propose a novel two-stage animal sound generation scheme based on a class-conditional GAN for data augmentation in ASC. During data generation, our DualDiscWaveGAN effectively generated virtual data from several classes of animal sounds by considering both the waveform and spectrogram of the sound data. Then, we calculated the confidence scores of the virtual data and selected the data with high scores for the augmentation. Through various experiments using two animal sound datasets, we demonstrated the effectiveness of the proposed scheme. The experimental results showed that the generative performance of the proposed scheme in FID and NDB, for all the classes or individual classes, surpassed that of the other conditional GANs. Specifically, our DualDiscWaveGAN exhibited scores of 26.45 and 43.82 in FID and averaged Intra-FID, respectively. In addition, the proposed scheme improved the baseline ResNet model by more than 0.8% and 13.4% in accuracy for the bird sound dataset and the frog sound dataset, respectively, compared to the second-best method.

The proposed scheme showed an excellent performance for a small number of classes. For delivering a more robust performance, the proposed scheme needs to be improved further to enable the analysis of more classes that are commonly encountered in the real world. Hence, in the future, we will investigate more advanced architectures of conditional GANs that can effectively generate sounds, even for a large number of sound classes.

## Figures and Tables

**Table 1:** Configurations of the comparative augmentation methods.

Method	Data Type	Augmentation Setting
Time stretching	Waveform	Speed scaling by a random value [0.8, 1.2]
Pitch shifting	Waveform	Pitch shifting by a random value [−2, 2]
Background noise addition	Waveform	Background noises from Freesound web DB
White noise addition	Waveform	Random noises with uniform intensity
Frequency masking	Spectrogram	Random mask size (max. 50% of frequency range) and index
Time masking	Spectrogram	Random mask size (max. 50% of time range) and index

Data type denotes the augmented data form. Augmentation setting presents the parameters or noise types used for the augmentation.

**Table 2:** FID and NDB for all the class data evaluations.

Conditional Generative Model	FID	NDB_all-classes_ (*K* = 100)
cGAN	31.73	33
ACGAN	37.96	22
Projection-cGAN	35.94	21
DualDiscWaveGAN	**26.45**	**4**

*K* indicates the number of bins. Bold values indicate the best score.

**Table 3:** Intra-FID and NDB for the class-wise data evaluations.

Class	Intra-FID/NDB_class-wise_ (*K* = 20)			
	cGAN	ACGAN	Projection-cGAN	DualDiscWaveGAN
*Cyanocitta cristata*	74.30/2	73.89/3	64.41/**1**	**60.59/1**
*Melospiza melodia*	44.26/4	52.20/9	**26.38/3**	28.05/**3**
*Cistothorus palustris*	18.35/4	17.07/**0**	**16.94**/1	18.15/1
*Geothlypis trichas*	76.60/6	63.83/10	63.15/4	**29.67/3**
*Spizella passerine*	87.85/6	101.52/7	128.06/9	**35.32/0**
*Setophaga aestiva*	96.70/5	116.30/7	126.61/6	**94.08/2**
*Corvus brachyrhynchos*	17.33/3	14.22/4	26.01/**2**	**11.98/2**
*Bombycilla cedrorum*	118.12/2	156.83/8	125.73/4	**116.30/1**
*Haemorhous mexicanus*	30.73/4	48.77/11	55.53/7	**18.31/1**
*Passerina cyanea*	45.06/5	123.38/4	62.40/5	**25.72/2**
Average	60.93/4.1	76.80/6.3	69.52/4.2	**43.82/1.6**

*K* indicates the number of bins. Bold values indicate the best score. Underlines indicate the second-best score.

**Table 4:** Performance comparison of the augmentation methods based on the NA birds dataset.

Method	Accuracy	Precision	Recall	F1-Score
ResNet	95.4	95.5	95.3	95.4
Time stretching	97.2	97.2	97.2	97.2
Pitch shifting	97.6	97.6	97.6	97.6
White noise addition	96.8	96.8	96.8	96.8
Background noise addition	-	-	-	-
Frequency masking	97.0	97.0	97.0	97.0
Time masking	94.4	94.5	94.4	94.4
Proposed scheme w/o DS	97.8	97.8	97.9	97.8
Proposed scheme	**98.4**	**98.4**	**98.5**	**98.4**

Bold values indicate the best score. Underlines indicate the second-best score. DS indicates data selection.

**Table 5:** Comparison of augmentation methods based on the SK frogs dataset.

Method	Accuracy	Precision	Recall	F1-Score
ResNet	65.8	77.0	73.8	69.5
Time stretching	62.8	78.4	69.9	65.7
Pitch shifting	53.8	66.2	61.2	56.1
Background noise addition	68.9	76.2	75.8	69.8
White noise addition	70.7	78.4	**79.1**	75.1
Frequency masking	65.2	68.7	72.4	61.9
Time masking	67.6	74.9	74.4	68.3
Proposed scheme w/o DS	83.7	82.0	76.0	76.6
Proposed scheme	**84.1**	**82.5**	76.3	**76.9**

Bold values indicate the best score. Underlines indicate the second-best score. DS indicates data selection.

**Table 6:** Ablation study of the components used in the proposed scheme for the SK frogs dataset.

Method	Accuracy	Precision	Recall	F1-Score
ResNet (No augmentation)	65.8	77.0	73.8	69.5
cGAN	74.6	77.9	75.8	74.1
cGAN with SD	83.7	82.0	76.0	76.6
cGAN with SD & DS (Proposed)	84.1	82.5	76.3	76.9

SD indicates spectrogram discriminator. DS indicates data selection.

## Data Availability

The dataset for North American bird species used in this study is publicly available at https://doi.org/10.5281/zenodo.1250689 (accessed on 10 September 2022). The dataset for South Korean frog species and the model used in this study are available on request from the corresponding author.
